# Antioxidant and Anticancer Activities of Heart Components Extracted from Iraqi *Phoneix Dactylifera* Chick

**DOI:** 10.31557/APJCP.2021.22.11.3533

**Published:** 2021-11

**Authors:** Mustafa F Hameed, Ihsan A Mkashaf, Ali A A Al-Shawi, Kawkab A Hussein

**Affiliations:** 1 *Ministry of Education, General Directorate of Education in Basrah, Basrah, Iraq. *; 2 * Department of Chemistry, College of Education for Pure Sciences, University of Basrah, Basrah, Iraq. *

**Keywords:** Apoptosis, breast cancer, cell cycle, microwave-assisted extraction, heart of Phoenix dactylifera

## Abstract

**Background:**

Breast cancer is one of the most frequent malignancies in women, and it is a major cause of cancer death worldwide, as well as one of the leading causes of cancer mortality. Traditional herbal therapy has been widely used in some developing countries as a complementary and alternative technique. Because of their low toxicity, medicinal edible plants have been allowed to minimize the risk of breast cancer and other diseases. The heart of Phoenix dactylifera is a well-known, safe, and common edible part of the *P. dactylifera* plant (Hilawi variety). The biological properties of heart of *P. dactylifera* are unclear, and the appeal warrants further investigation. The aim of this study is to look into the chemical compositions, antioxidant and anticancer properties of heart of *P. dactylifera* extract obtained via microwave-assisted extraction. Methods: Microwave-assisted extraction, ethanol solvent, gas chromatography–mass spectroscopy (GC-MS) analysis, DPPH assay, MTT assay, acridine orange/ethidium bromide staining, cell cycle, reactive oxygen species, and apoptosis were all used to evaluate the activity of heart of *P. dactylifera*.

**Results:**

GC–MS was used to identify the chemical compositions of heart of *P. dactylifera* extract, which revealed about 15 bioactive compounds. The antioxidant activity of heart of *P. dactylifera* extract was determined to have an IC_50_ value of 114.2 µg/ml. The cytotoxicity was measured using MCF-7 cells, and the IC_50_ was reported to be 620.1 µg/ml. The cell cycle was arrested at the G1 gate, resulting in the formation of reactive oxygen species and apoptosis. Conclusion: The findings suggested that regular consumption of *P. dactylifera* heart components is important for nutrition and immune system support in the prevention of breast cancer, and that more research into molecular apoptotic pathways is needed.

## Introduction

Many tree species, including coconuts, area nuts, and date palms, are members of the Arecaceae family (Phoenix dactylifera). The *P. dactylifera* tree is a well-known member of the genus, with over 3,000 different varieties found throughout the world (Khierallah et al., 2017). Some of the tree parts used in traditional medicine are the flowers, date fruits, heart, date seeds, and date skin (Al-alawi et al., 2017). Dates vary in shape, size, weight, color, sweetness level, and price depending on location (Hussam et al., 2016). In Iraq, there are approximately 600 different varieties of the *P. dactylifera* tree, distributed based on the soil type and the effects of the climate (Kareem et al., 2018). One of the most well-known Iraqi dates is the Hilawi variety, it is a well-known among farmers, so it’s easy to distinguish it from other *P. dactylifera* trees. Production of this variety fell to 22,300 tons in 2003 due to a lack of attention to this form of date, massive sale of the better varieties during wars, urban growth, and emigration of farmers out of the region. The value of the Hilawi variety stems from its resistance to climate, aridity, and frost, as well as its sweet flavor and early maturity (Bastway et al., 2008). Some date tree parts, such as fruits, pollen, and leaves, have been studied for their biological functions in Egypt and Saudi Arabia (Khan et al., 2016; Bentard et al., 2017). Furthermore, when compared to other extraction methods, microwave-assisted extraction is a selective, fast, and low-cost method for determining the chemical composition of medicinal herb extracts. As a result, controlling the MAE extraction process by the use of a suitable solvent, time, and heat would improve MAE selectivity, making it a suitable method for extracting medicinal herb components for biological functions and applications in medicine (Lin et al., 2012). Different countries have studied the chemical composition of date palm and classified it as flavonoids, phenolic acids, carotenoids, phytosterols, and phytoestrogens, all of which have different biological activities (Mohammed et al., 2017; Nehdi et al., 2018; Maqsood et al., 2020). A clinical study on pediatric cancer discovered that *P. dactylifera* palm date (Ajwa) had a positive impact and could improve future care (Soad et al., 2019). Other branches of the phoenix tree have been studied in the past to see whether they can help prevent breast cancer (Najla et al., 2017). According to numerous Iraqi research, this has resulted in an increase in breast cancer mortality during the previous few years. This increasing of mortality could be attributed to environmental changes and dangerous food systems (Alwan, 2016; Mjali et al., 2021; Mjali et al., 2020). Chemotherapy treatment for breast cancer or other cancer diseases will increase the patients side effects (Mjali et al., 2018). As a result, non-toxic and edible medicinal plants may be taken on a regular basis to prevent the development of breast cancer and avoid the difficulties associated with chemotherapy (Ümit et al., 2017). The goal of this study was to identify the chemical compositions, antioxidant and anticancer effects of an ethanol extract of *P. dactylifera* heart (Hilawi variant) prepared using the microwave-assisted extraction (MAE) method.

## Materials and Methods

On April 1, 2019, collected heart of *P. dactylifera* (Hilawi variety) from a farm in the southern region of Basrah province, Abo-AlKhaseeb district, south of Iraq. The species of Hilawi variety was classified by Dr. Suhad Abdulsada, University of Basrah, College of Science, Environmental Department. The heart was peeled, cut, mechanically dried, and ground before being stored at 4 oC. The research was conducted from March to December 2019. 

Heart of *P. dactylifera* extraction method using MAE

In a round conical flask, combine 5 g of heart with 100 ml of 99% ethanol solvent, stir thoroughly, and let for 20-30 minutes in a microwave-assisted extraction homemade. For 5 minutes, the extraction process was initiated at a temperature of 50°C. The extracts were allowed to cool for 10-20 minutes before being centrifuged at 3,000 rpm for 15 minutes. They were then filtered using 20 m of Whatman filter paper and kept at 4°C. (Hameed and Al-Shawi, 2020). 

*GC-MS analysis method of heart ethanol extract*

Gas chromatography mass spectrometry was used in the study. A GC that works in conjunction with the Mass Hunter workstation software (Agilent 7890B GC with 5977A MSD, USA). The column temperature gradient began at 40 °C on a phenyl methyl siloxane 5 percent column under 6.0799 psi pressure. The temperature was raised from 50 to 280°C at a rate of 10°C/min, resulting in a linear gradient. After the solvent was switched off, the injector was kept at 290°C for 4 minutes. Helium was used as the carrier gas at a flow rate of 1 ml/min. Pulsed splitless injection with a molecular weight test range of 35-650 m/z and a test rate of 1,562 (N2). The extract was filtered with syringe filters before being injected onto the GC column and analyzed with the NIST library.

*Antioxidant activity method*

The antioxidant activity of *P. dactylifera* heart extracted by MAE was determined using the simple DPPH (2,2-Diphenyl-1-picrylhydrazyl) technique (Young et al., 2013). Different concentrations of HPD (10, 20, 30, 40, 50, 60, 70, and 80) µL were added to 200 µL of DPPH (1 mm) in a 96 well/plate and left in a dark place for 30 minutes. Determine the absorbance at 490 nm using a micro-plate reader (ELISA, Asyshitech., UK). HPD antioxidant activity was calculated using Equation (1):

% Antioxidant activity = {1- [ A (HP+DPPH)/ A(DPPH)]} x 100                                    (1)

*Anticancer activity of heart of P. dactylifera*

*MTT assay*

The Iraqi national center for cancer research, University of Al-Mustansriah, Baghdad, Iraq, provided the human breast cancer cell MCF-7. The MTT test was used to calculate the IC_50_ value for HPD. MCF-7 cancer cells were grown in 96-well plates supplemented with RPMI-1640 at a density of 1x104 cells per well. Cells were treated for 24 hours with 100 µL of complete culture media containing (400, 800, 1,000, 1,200, 1,400, 1,400, 1,800) µg/mL of HPD with an equivalent amount of DMSO as a negative control. Following a 24-hour incubation period to determine cell viability, 10 µL of MTT (5 mg/mL) in phosphate buffered saline was administered to each well and incubated for four hours. After removing the medium, 150 µL DMSO was gently shaken into each well, and the absorbance at 620 nm was measured with a micro-plate reader (ELISA, Asyshitech., Manchester, UK) (Mohammad et al., 2019).

*AO/EB staining*

Trypsin fersin was introduced to MCF-7 cells through trypsinyzation, followed by medium RPMI-1640, a clean and sterilized slide was placed on the planted cells dish, and 5000 MCF-7 cells were planted on the slide cover, which was then securely covered by the parafilm sheet for 24 hours in the incubator (5% CO2 and 37^o^C). After 24 hours, discard the medium and replace it with the HPD IC_50_ value. Tightly shut the dish and re-incubate for another 24 hours. After that, remove the slide cover and place it in a clean slide with 70 µL of AO/EB stain before photographing the slide with a fluorescence microscope (Flourecent Microscope, Zeiss axiolabe, CE, Germany) (Al-Shawi et al., 2021).

*Flowcytometry analysis*

*Cell Cycle arrest*

The G1, S, and G2 stages were detected by flow cytometry (BD biosciences, San Jose,CA, USA). MCF-7 cells were treated with HPD / values for 48 hours. The cells were then rinsed in PBS and fixed in 70% ice-cold ethanol overnight at 4°C. Cells were stained for 30 minutes in the dark at room temperature with a solution containing 50 µg/mL PI and 100 µg/mL RNase A after being washed twice with PBS (Al-Shawi et al., 2020).

*Reactive oxygen species ROS*

The variations in the production of intracellular reactive oxygen species were studied (DCFH-DA). MCF-7 cells were cultured in 6 well culture plates overnight. After 48 hours of treatment with or without the IC_50_ value of HPD, the cells were treated with 10 mol/L DCFH-DA for 30 minutes at 37°C. In the positive control group, cells tagged with DCFH-DA were treated with 1 µL rose up for 30 minutes. Cells were then collected, washed, and re-suspended in PBS before being flow cytometrically examined for 2,7-dichlorfluorescein (DCF) fluorescence (Khan et al., 2012).

*Apoptosis*

HPD IC_50_ values were applied to MCF-7 cells for 48 hours. The cells were collected, washed twice with PBS, and tagged with 5 uL FITC-conjugated annexin V, as directed by the manufacturer. After being incubated in the dark for 10 minutes and then labeled with PI, the samples were immediately examined on a flow cytometer (BD biosciences, San Jose,CA, USA) (Al-Shawi et al., 2020).

*Statistical analysis*

The DPPH and MTT approaches were tested three times. GraphPad Prism 8.1 for Windows was used to analyze the results.

## Results

*GC-MS analysis of P. dactylifera heart*

The GC–MS analysis of the ethanol extract of heart produced by MAE revealed around 15 compounds, with seven high peaks among them (indicated with red color). According to NIST library, the first peak was compound 6 (cis-vaccenic acid , RT = 24.092 min) followed by compound 5 (n-hexadecanoic acid; RT = 22.492 minutes), compound 10 (hexadecanoic acid, 2-hydroxy-1-(hydroxymethyl) ethyl ester; RT= 27.087 minutes), compound 13 (Squalene; 29, RT= 477 minutes), compound 1 (3-amino-2-oxazolidinone; RT= 5.463 min), compound 7 (octadecanoic acid; RT= 24.301 minutes), compound 11 (9,12-Octadecadienoic acid (Z,Z)-, 2-hydroxy-1-(hydroxymethyl) ethyl ester, RT= 28.428 minutes), (Table 1). 

*DPPH assay of P. dactylifera heart*

**Figure 1:**
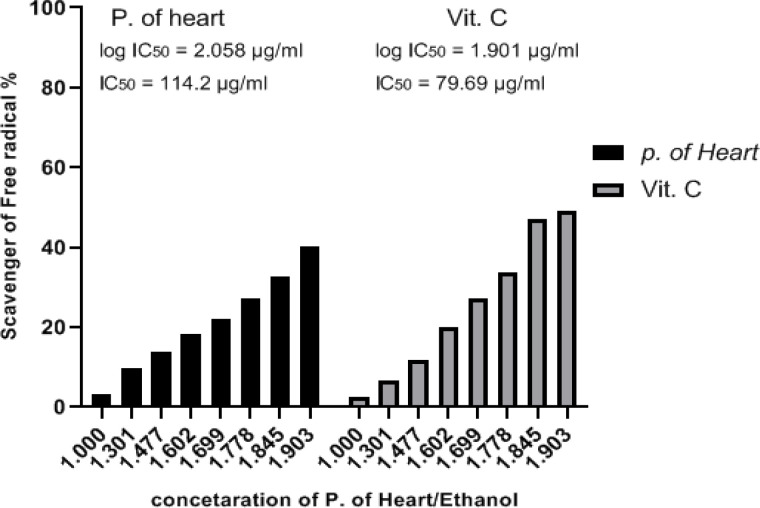
In Comparison to Vitamin C (IC_50_ = 79.69 µg/ml), the Scavenger Activity Percent of HPD Extract (IC_50_ = 114.2 µg/ml) was shown

It’s the first study of its kind to look at the antioxidant activity of heart of *P. dactylifera* chick (Hillawi variety) extracted by MAE using a cheap DPPH process. Various concentrations of heart extract (0-80 µl) were combined with DPPH for 30 minutes and the absorbance at 490 nm was measured. The color shift of DPPH from dark violet to translucent suggested antioxidant activity. When compared to vitamin C (IC_50_ = 79.69 µg/ml) as a positive control, the IC_50_ value was 114.2 µg/ml (Figure 1). 

*Anticancer evaluation of P. dactylifera heart*

*MTT assay*

**Figure 2:**
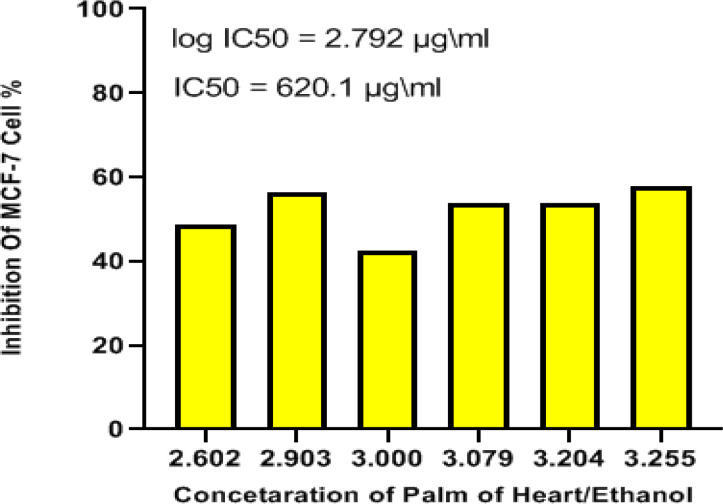
Graph Pad Prism 8.1 was Used to Perform an MTT Assay of HPD Extract against MCF-7 Cancer Cells at Different Concentrations and Calculate the IC_50 _Value

MTT assay was used to investigate the cytotoxic effect of heart extract on MCF-7. Regardless of concentration, a decrease in cell viability occurred in a time-dependent manner for concentrations showing the extract efficacy as compared to the untreated control. The observation would imply that the extract has a higher action on the MCF-7. After 24 hours, the IC_50_ value was observed as 620.1 µg/ml (Figure 2). Thus, before flow cytometry analysis, the morphological changes of MCF-7 cells treated with the IC_50_ value of heart extract were recorded using an optical microscope (Figure 4).

*Acridine orange/ethidium bromide staining*

**Figure 3:**
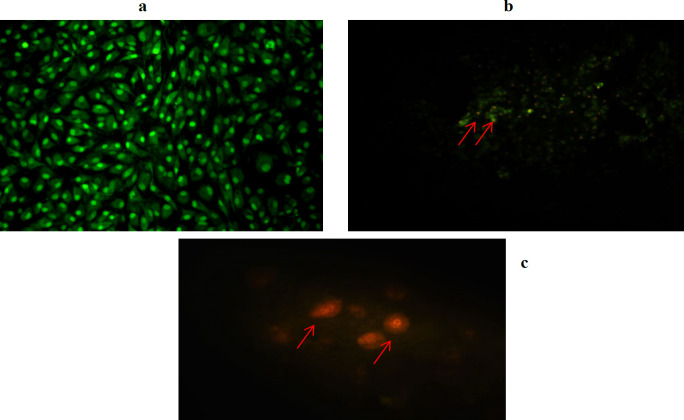
MCF-7 Cancer Cells Stained with AO/EB Revealed: a, untreated cells (10x); b, Cells treated with HPD (10x); c, Cells treated with HPD (40x).

Acridine orange/ethidium bromide (AO/EB) fluorescent staining, when viewed under a fluorescent microscope, can be utilized to reveal apoptosis-associated alterations in cell membranes. This approach can also distinguish between cells in various phases of apoptosis. Acridine orange is a vital dye that will stain both live and dead cells, whereas ethidium bromide will only stain cells that have lost membrane integrity, making live cells appear consistently green. To improve heart of palm anti-breast function, AO/EB staining was employed to detect apoptosis and DNA damage (red arrows) in MCF-7 cells. The IC_50_ concentration of heart extract was compared to untreated control cells. Cells that were not treated displayed a clear green hue, indicating that they were alive, but cells that were treated with heart extract displayed apoptosis and DNA damage (Figure 3 (panels a, b and c).

*Flow cytometry analysis*

*Cell cycle*

Researchers have shown a growing interest in using cell cycle as a new method for cancer therapy in the last decade. It is well documented, for example, that there is a link between cell cycle advancement and prevention of cell proliferation and apoptosis in cancer cells. To analyze cell distribution in different stages of the cell cycle, we used flow cytometric studies in conjunction with DNA staining with PI. Our findings show that treating MCF-7 with the IC_50_ value of heart extract results in an increase in the number of cells at G2 (11.95%) and S (35.09%) phases in treated cells compared to control (G2 (10.53%), and S (30.77%), while G1 (42.18%) phase in treated cells was lower than control (G1 (46.59%)), Figure 5(A and B). As a result, MCF-7 cells were arrested in the G1 phase.

*Reactive oxygen species ROS*

DCFH-DA, a fluorescent probe, was utilized to detect the quantity of ROS generation in order to explore the role of reactive oxygen species in inducing apoptosis using IC_50_ value of heart extract. Figure 5 (C and D) show an increasing trend in intracellular ROS in MCF-7 cells in the presence of heart extract (DCFH, 78.1%) compared to the control (DCFH, 98.8%). This behavior could indicate that heart extract stimulation of apoptosis in MCF-7 breast cancer cells is linked to ROS generation.

*Apoptosis*

We used flow cytometry after annexin V-FITC/PI double labeling to gain a better understanding of the cytotoxicity mechanism of the heart extract. The early and late apoptosis values correlate to MCF-7 cells in the presence of IC_50 _value of heart extract after 48 h relative to the control sample (untreated cells) were computed using the data shown in Figure 5 (E and F). The control cells showed early apoptosis (Q1= 4.77%), late apoptosis (Q2=3.59%), necrosis (Q3=3.09%), and living cells (Q4= 89.6%), whereas the treated cells showed early apoptosis (Q1= 13.0%), late apoptosis (Q2=10.1%), necrosis (Q3=6.9%), and live cells (Q4= 62.7%). As a result, the discovery may lead us to conclude that apoptosis is the mechanism by which heart extract causes cytotoxicity in MCF-7 cells.

**Table 1:** The Highest Peaks Ratio of Chemical Compositions of HPD Extracted by MAE Process, as Well as Their Functions, were Analyzed Using GC-MS

No	RT in min	Name of compound	Functions
1	5.463	3-Amino-2-oxazolidinone	A nitrofuran metabolite, which is bound to the tissues can be determined, using monoclonal enzyme-linked immunosorbent assay(ELISA) method.
2	5.852	Ethanol, 2-(trimethylsilyl)-	* Protecting reagent for carboxyl and phosphate groups. *Used to synthesize Teoc-protected amines via alcoholysis of the corresponding isocyanates.
3	10.10	Ethyl cyanoacetate	
4	14.19	Cyclohexanone, 2-(2-butynyl)-	It was used to investigate the Knoevenagel condensation reactions in microreactor using zeolite catalysts obtained by grafting amino groups onto NaX and CsNaX zeolites.
5	22.47	n-Hexadecanoic acid	Organic synthesis
6	24.09	cis-Vaccenic acid	*A fatty acid that is found naturally in animals and plants and also can be created in laboratory settings.
7	24.30	Octadecanoic acid	* Widely used in a variety of applications, including personal care products and cosmetics.
8	24.42	1-Heptatriacotanol	Has been associated with diverse health benefits such as:
9	25.96	9-Octadecenamide, (Z)-	*Cardiovascular diseases,
10	27.08	Hexadecanoic acid, 2-hydroxy-1-(hydroxymethyl)ethyl ester	*Cancer,
11	28.42	9,12-Octadecadienoic acid (Z,Z)-, 2-hydroxy-1-(hydroxymethyl) ethyl ester	*Immune function and inflammation.
12	28.48	Oleic Acid	
13	29.47	Squalene	Used in hardening soaps, softening plastics and in making cosmetics, candles and plastics
14	34.50	Diosgenin	Has anti-hypercholesterolemic effects
15	35.05	gamma.-Sitosterol	* Has a variety of industrial uses including as a slip agent, a lubricant, and a corrosion inhibitor.

**Figure 4:**
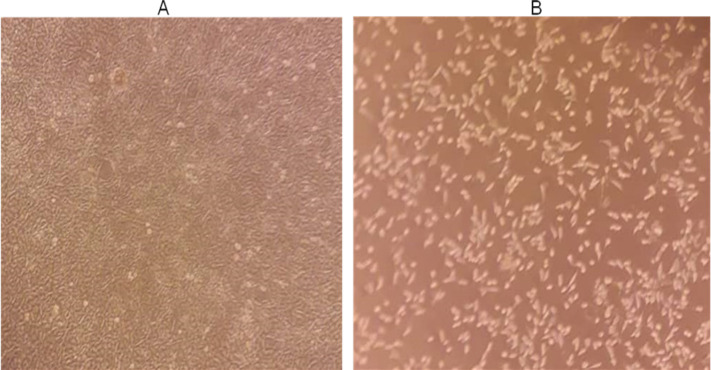
The IC_50_ Value of HPD Extract has an Impact on the Morphology of MCF-7 Cells

**Figure 5:**
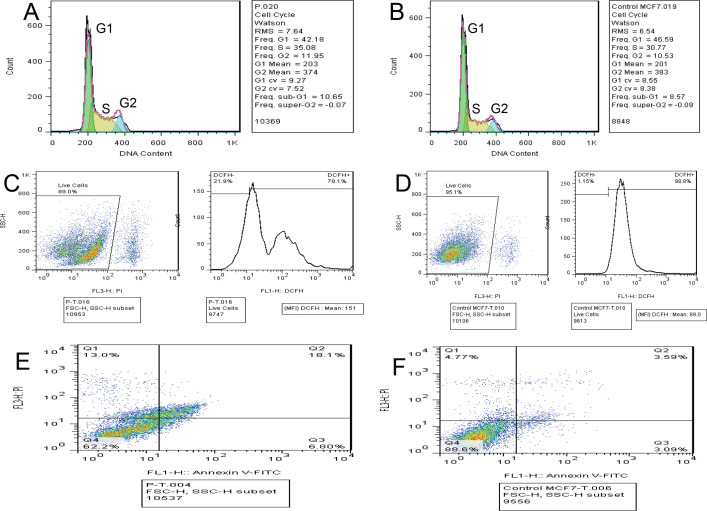
Flow Cytometry Analysis. A, Cell cycle (treated cells with IC_50_ value of HPD extract); B, Cell cycle (Untreated cells); C, Reactive oxygen species (treated cells with IC50 value of HPD extract); D, Reactive oxygen species (Untreated cells); E, Apoptosis (treated cells with IC_50_ value of HPD extract); F, Apoptosis (Untreated cells)

## Discussion

The chemical composition of the *P. dactylifera* heart is affected by soil type, environmental influences, extraction procedures, and the site of *P. dactylifera* chick tree growth. The Hilawi variety is one of the most abundant in Iraq, and the chemical composition analysis, antioxidant, and anticancer properties had never been described previously. It was straightforward and quick to develop microwave-assisted extraction as a selective approach for ethanol-based extraction of *P. dactylifera* heart components.

Aqueous ethanol extract of *P. dactylifera* heart was employed in a study to analyze the polyphenols component using GC-MS. The study proved the worth of *P. dactylifera* heart extract by discovering that an ethanol extract of the heart might avoid cardiotoxicity and nephrotoxicity (Sahyon and Al-Harbi, 2020). Using heart extract and GC-MS analysis, we found 15 bioactive chemicals in our study. Some of these chemicals have health benefits, such as cis-vaccenic acid (used in cardiovascular disease, cancer, immunological function, and inflammation) (Tripathy and Jump, 2013), and squalene (the principal therapeutic use of squalene is now as an adjuvant therapy in a variety of cancers) (Jun et al., 2021). 

Hence, antioxidants found in medicinal plants can delay or prevent the oxidation of lipids or other molecules. Because several lipid-oxidation products have been shown to interact with biological components and induce cellular damage, the oxidation process has been linked to chronic disorders such as cancer. Polyphenols are the most important plant chemicals with antioxidant action. These are found in most plants and are thought to protect against free radical-related damage in a variety of methods, including direct scavenging of free radicals and inhibition of enzymes involved in free radical generation. Spices and herbs are high in antioxidants. They have been used to improve flavor, aroma, and color in food and beverages. Spices and herbs have also been utilized to treat several ailments due to their high antioxidant activity. (Xu et al., 2017; Hassan et al., 2017).

Some portions of the *P. dactylifera* tree were discovered to be essential in antioxidant studies; for example, dates are regarded as a good source of antioxidants due to carotenoids and phenolics (Siddiqi et al., 2020). Several studies have proven the usefulness of *P. dactylifera* as a natural antioxidant. Quarda et al., (2019) discovered that date seeds from the *P. dactylifera* L. plant may be a good source of antioxidant and enzyme inhibitory bioactive substances. Tahir et al., (2019) discovered that paneer supplemented with date extracts improved the antioxidant activity of the date extracts. Hamutal et al., 2015) discovered that date phenolics and flavonolics from the Amar and Hillawi kinds showed a tight structural action as antioxidants and anti-atherogenics. In this work, we discovered that *P. dactylifera* (Hillawi variety) heart extract has good antioxidant activity when tested with DPPH, when compared to vitamin C as a positive control. The scavenging effect of cardiac extract may be attributable to chemical composition-related actions (the hydroxyl group plays a major role in antioxidant scavenging levels). As a result, the phenolic and flavonoid ratio in heart extracts may have antioxidant characteristics.

On the other hand, cancer has been an ongoing battle around the world, with a lot of progress in cures and preventative therapies. Chemotherapy, radiation, and chemically derived medications are currently used as therapies. Chemotherapy, for example, can put people under a lot of stress and harm their health. As a result, there is an emphasis on employing alternative cancer treatments and therapies. Herbal medicines have been utilized and continue to be used as the primary source of medical treatment for many years. Plants have been utilized in medicine for centuries because of their natural antibacterial characteristics. As a result, research has focused on researching the possible qualities and applications of terrestrial plant extracts for the development of potential based medications for diseases such as cancer (Abu-Darwish and Efferth, 2018). Many plant species are already being used to cure or prevent cancer growth. Multiple studies have identified plant species having anticancer characteristics, with a particular emphasis on those utilized in herbal medicine in developing nations. Plants that are utilized in cancer treatment include ginko, goldenseal, ginseng, garlic, Echinacea, aloe vera, and saw palmetto (Mayzlish-Gati et al., 2018). Furthermore, during the period of medicinal plant treatment for breast cancer, a great variety of plants and their substances have been reported to demonstrate promising anticancer actions against breast cancer in both in vivo and in vitro models. However, because to a dearth of randomized clinical trials, their therapeutic benefits on breast cancer treatment remain uncertain (Baraya et al., 2017; Aumeeruddy and Mahomoodally, 2019).

Phoenix dactylifera have been shown to have anticancer properties in some studies. Dalia and Sahar discovered that phoenix dactylifera seeds can protect rats from CCL4-induced liver toxicity (Dalia and Sahar, 2014). Reem et al., (2020) found that date palm fruit has chemopreventive properties against pancreatic cancer. Muqtadir et al., (2018) discovered that ethyl acetate of Ajwa date inhibited prostate cancer and induced apoptosis through the S phase. Khan et al., (2018) found that Ajwa date aqueous extract improves liver functions and prevents hepatocellular carcinoma. Some studies showed the cytotoxicity of date palms against breast cancer, Hanen et al., (2018) used aqueous ethanolic extract of parthenocarpic dates and found that it prevented the growth of MCF-7 and MDA-MB-231 breast cancer cells. Nael et al., (2018) found that an ethyl acetate extract of *P. dactylifera* L. has potent anti-proliferative effects on MCF-7 cell. Khan et al., (2016) found that the methanolic extract of Ajwa date inhibited MCF-7 cell by inducing apoptosis and cell cycle arresting in S phase. 

For the first time, we examined the inhibitory action of *P. dactylifera* (Hillawi variety) heart extract on MCF-7 cancer cells in our study. The IC_50_ value obtained after 24 hours of treatment was 620.1 µg/ml, suggesting the efficiency of heart extract in suppressing MCF-7 cell growth. The IC_50_ value was utilized to evaluate the anticancer capabilities further. The effect of IC_50_ value of heart extract on MCF-7 has been demonstrated by AO/EB through damage of cells stained with red color, compared to living cells labeled with green color (Liu et al., 2015). The IC_50_ value and AO/EB staining indicated that the heart extract had a moderate effect. Flow cytometry study (Annexin-V/FITC and PI staining) indicated apoptosis, which was enhanced by measuring early and late apoptosis. It was observed that treated cells have a higher percentage of late apoptosis than untreated cells. It was demonstrated that apoptosis is involved in the process underlying the interaction of heart extract and MCF-7 cells, indicating that further research is needed to understand the target apoptotic pathway. In the current study, we observed a considerable increase in G1 phase, indicating an increase in apoptotic cell death. A high level of ROS can cause oxidative damage of cellular proteins, lipids, and nucleic acids, ultimately triggering the cellular death process via apoptosis or necrosis. Chemotherapeutic drugs frequently include a mitochondrial apoptotic route mediated by ROS generation. As a result, innovative therapeutic plant extracts that regulate the buildup of intracellular ROS may be considered a promising strategy to tumor growth. In our study, we observed that the selective growth inhibition of MCF-7 caused by the heart extract is linked to the apoptosis pathway via ROS generation. As a result, it appears reasonable to assume that heart extract might be utilized to prevent breast cancer by regulating daily dose. It is the first study to use microwave-assisted extraction to assess the antioxidant and anticancer activities of *P. dactylifera* (Hillawi variation) heart extract. 

In conclusion, microwave-assisted extraction is a very simple extraction method that requires only a small set of inexpensive instruments and has a selective active role in the heart of *P. dactylifera* extraction process with ethanol solvent. Furthermore, GC–MS study of heart extract yields a mini-library of bioactive compounds with antioxidant and anticancer properties. By reducing ROS and late apoptosis, the heart extract arrests MCF-7 cells in the G1 phase. The findings prompted us to isolate and classify the molecules responsible in the heart of *P. dactylifera*, as well as investigate their mechanistic targets. As a result, the edible parts of the *P. dactylifera* tree are rich in bioactive compounds and should be a mainstay of our bodies good nutrition systems, consumed on a regular basis to avoid breast cancer and other diseases.

## Abbreviation

HPD (heart of *P. dactylifera*), IC_50_ (inhibitory concentration 50%), MCF-7 (Michigan Cancer Foundation-7), NIST (National Institute of Standards and Technology), RPMI (Roswell Park Memorial Institute), RT (retention time).

## Author Contribution Statement

Dr. Ali A. A. Al-Shawi worked on the design, implementation of project, analysis of results, preparation of the manuscript, writing and editing. Mustafa F. Hameed and Ihsan A. Mkashaf worked on the DPPH experiment. Kawkab A. Hussein worked on GC-MS results analysis. 

## Ethical statement

The ethics committee by University of Basrah, College of Education for Pure Science, Chemistry Department, Iraq, approved the project ethically. 

## Conflict of interest

The authors declare that there is no conflict of interest.
